# Can Dental Microwear Textures Record Inter-Individual Dietary Variations?

**DOI:** 10.1371/journal.pone.0009542

**Published:** 2010-03-04

**Authors:** Gildas Merceron, Gilles Escarguel, Jean-Marc Angibault, Hélène Verheyden-Tixier

**Affiliations:** 1 UMR CNRS 5125, Paléoenvironnements et Paléobiosphère, Campus de La Doua, Université Claude Bernard Lyon-1, Villeurbanne, France; 2 Laboratoire Comportement et Ecologie de la Faune Sauvage, INRA, BP 52627, Castanet Tolosan, France; University of Toronto, Canada

## Abstract

**Background:**

Dental microwear analyses are commonly used to deduce the diet of extinct mammals. Conventional methods rely on the user identifying features within a 2D image. However, recent interdisciplinary research has lead to the development of an advanced methodology that is free of observer error, based on the automated quantification of 3D surfaces by combining confocal microscopy with scale-sensitive fractal analysis. This method has already proved to be very efficient in detecting dietary differences between species. Focusing on a finer, intra-specific scale of analysis, the aim of this study is to test this method's ability to track such differences between individuals from a single population.

**Methodology/Principal Findings:**

For the purposes of this study, the 3D molar microwear of 78 individuals from a well-known population of extant roe deer (*Capreolus caprelous*) is quantified. Multivariate statistical analyses indicate significant seasonal and sexual differences in individual dental microwear design. These are probably the consequence of seasonal variations in fruit, seed and leaf availability, as well as differences in feeding preference between males and females due to distinct energy requirements during periods of rutting, gestation or giving birth. Nevertheless, further investigations using two-block Partial Least-Squares analysis show no strong relationship between individual stomach contents and microwear texture. This is an expected result, assuming that stomach contents are composed of food items ingested during the last few hours whereas dental microwear texture records the physical properties of items eaten over periods of days or weeks.

**Conclusions/Significance:**

Microwear 3D scale-sensitive fractal analysis does detect differences in diet ranging from the inter-feeding styles scale to the intra-population between-season and between-sex scales. It is therefore a possible tool, to be used with caution, in the further exploration of the feeding biology and ecology of extinct mammals.

## Introduction

### Mammal Dental Microwear, Feeding Habits, Ecology, and Evolution

**Figure 1:**
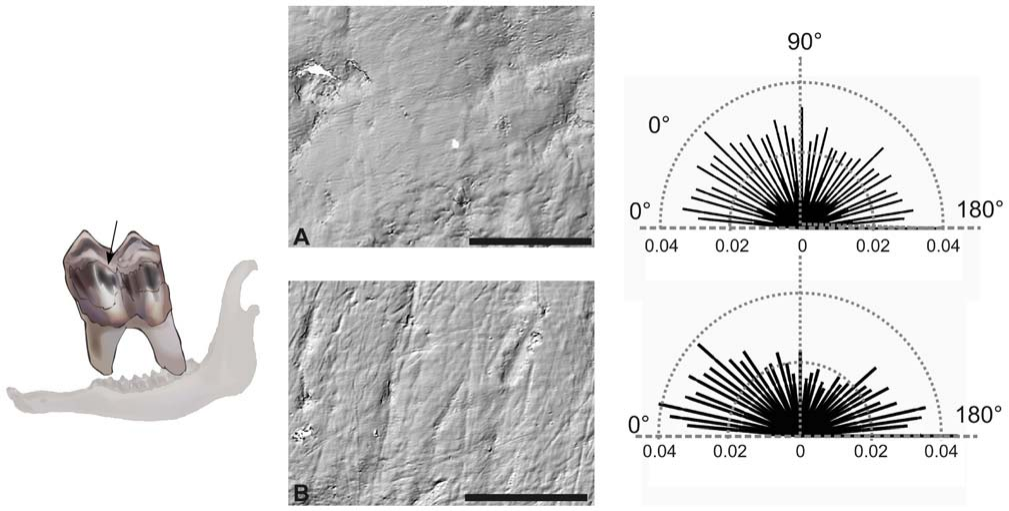
Molar microwear textures reflect differences in feeding behaviors between males and females. The dental microwear texture is captured as a 3D virtual surface on shearing molar facets, here noted with an arrow [42]. Photo-simulations of the microwear surface of two individuals and the corresponding rosette plot of relative lengths taken at 36 different orientations from a male (A; INRA 013) and a female both slaughtered in winter (B; INRA 001). Scale bars: 50 µm.

Over the past three decades, analyses of dental microwear have been widely used for characterizing the feeding habits of extinct mammals [1], [2], [3]. Dental microwear, the study of the microscopic use-wear scars left in the enamel (Figure 1), provides direct information about what an individual ate over a period of time in the past [4]. For instance, the proportion of browsing and grazing can be estimated from microwear pattern for ruminant species that became extinct millions of years ago [1], [5]. Clearly, differences in the abundance of silica phytolith between monocotyledons and dicotyledons [6] are sufficiently important to be detected by dental microwear analysis. This opens the possibility of a fossil species feeding habit comparison from both palaeoecological/palaeoenvironmental and evolutionary points of view. Indeed, more than just an efficient way to determine the ecology of fossil species, reconstructing the diet of extinct mammals is a key-issue for deciphering ecological niche partitioning among species as well as for tracking long-term environmental changes [7], [8], [9], [10], [11], [12].

Because it provides short time-scale information ranging from a few days to a few weeks, the dental microwear texture is, at least theoretically, the most appropriate proxy for finding the fallback foods of fossil species. Nevertheless, in spite of many studies focused on the comparisons with present-day mammalian species [1], [13], [14], [15], [16], we still know precious little about how efficient dental microwear analysis really is in detecting such fallback foods. These food items, consumed seasonally (such as blackberries for the roe deer), actually drive the evolution of dental and mandibular morphologies and even digestive specializations more effectively than the preferred foods which are consumed almost daily all year round [17], [18]. Therefore, a better description of the feeding ecology of extinct species would undoubtedly improve our understanding of mammalian evolution. With this in mind, an ongoing challenge is now test whether or not the dental microwear design actually reflects intra-specific variations in diet, especially for species with a narrow spectrum of food preferences. The limitations of traditional microwear analysis techniques in detecting such variations are actually due to a lack of protocol repeatability [19] to potential measurement errors among and between observers [20]. Nevertheless, for a few years now an advanced methodology has provided repeatable, quantitative characterizations of 3D surfaces free of observer measurement errors (Figure 1) [9], [21], [22]. This method, named scale-sensitive fractal analysis (SSFA hereafter), appears to be a promising tool for the more detailed investigation of potential variations in dietary composition and the potential detection of variations in preferred and fallback foods.

### The Dourdan Forest Roe Deer Case Study

Parallel to the practical limitations summarized above, another constraint of the interpretation of microwear analysis results is the lack of reliable and independent information pertaining to the individuals being investigated. Indeed, most of the comparative data on present-day species was gathered from individuals whose date of death, location, environment, age and even sex remain unknown.

Here we specifically investigate the molar microwear textures of a wild population of roe deer, *Capreolus capreolus* (Cervidae, Ruminantia) from the Dourdan forest (Ile de France, France). The date of death, sex, and stomach contents were recorded for 120 individuals [23]. This gives us the opportunity to test how well the SSFA can detect dietary changes from season to season and differences in feeding habits between males and females. These seasonal and sexual variations in diet are due to the availability of fruits, seeds and foliage and to the differences in energetic requirements between males and females respectively. The combination of the two sets of data, dental microwear textures and stomach contents, allows us to perform individual-scale analyses in order to decipher whether or not some food items have an immediate impact on the enamel surface. This is especially true for fallback foods such as blackberries, which carry many millimetric seeds. These summer fruits represent few items, but are presumably hard enough to impact the enamel surface very quickly.

The natural history and ecological habits of the roe deer are relatively well known. This ruminant species appeared about 3 million years ago. It was primarily a forest dweller, then it adapted to a wide range of climatic variations and vegetation, including modern “cultivated landscapes”. Today roe deer occupy a wide variety of habitats: boreal, deciduous, coniferous and Mediterranean forests, moorland or farmland mosaic, agricultural plain and even suburban areas [24]. The feeding behavior of the roe deer is very flexible as they can eat leaves and buds of deciduous or coniferous trees as well as shrubs, forbs, ferns, grasses, cultivated plants, fruits and seeds in various proportions according to what is available, depending on their habitat and on the season [25], [26]. However for a given habitat and season, roe deer prefer a few items from the range of food available, and most particularly the “concentrate foods” rich in soluble carbohydrates [27]. Indeed the digestive anatomy of the roe deer has adapted to process food that meets its high energy and nutrient requirements [28]. Because wild seeds and fruits such as acorns [29] and the seeds of cultivated plants [28] are particularly rich in soluble carbohydrates, they can make up a large part of the diet when they are sufficiently available. In deciduous forests, the consumption of seeds and fruits, mainly acorns, peaks in autumn (17% of the diet on average in Europe) [29] but it is highly variable depending on mast abundance, reaching up to 89% of acorns in the diet in a mast-rich autumn [30]. In European deciduous forests, most of the diet is composed of brambles (*Rubus* sp), ivy (*Hedera helix*) and the leaves of trees throughout the year, with the addition of acorns when available in autumn and winter, blackberries in summer, and wild forbs in spring and summer [26].

These overall trends mask subtle inter-individuals variations mainly linked to sex, age and that can be evidenced only through detailed pluri-seasonal studies in the same area. Because males are slightly heavier than females or the young, and because nutritional requirements vary between sexes and seasons according to the differences in reproductive and growth investments, some changes in the dietary composition are expected to occur. Unfortunately as detailed studies of dietary composition require sufficient samples of rumen for each sex in all four seasons, they are very scarce. Most of the published studies of diet used samples obtained during the normal hunting season in autumn and winter [30], [31], [32]. The study of Cransac et al. [23] provides data to compare individual dietary compositions (rumen samples) between sexes and seasons in the deciduous forest of Dourdan (France). There, the main foods eaten by roe deer throughout the year were brambles (26–57% as percentage of dry matter weight in stomach contents) and the leaves of trees and shrubs (10–29% of dry matter weight). Various additional fallback foods were eaten: honeysuckle in spring (*Lonicera peryclimenum*, 10% dry matter weight), blackberries in summer (15% dry matter weight) and acorns in both autumn (about 60% dry matter weight) and winter (about 17% dry matter weight). Brambles are semi-deciduous bushes that provide foliage for the deer all year round in Dourdan. There is an abundance of acorns since oaks dominate the arboreal layer of the Dourdan forest [23]. These centimeter-scale seeds browsed on the ground by the deer differ in size from the millimeter-scale seeds found in their dozens per bramble fruit (blackberries). Cransac et al. [23] found significant dietary differences between the sexes during summer and winter: in summer, females eat more bramble leaves (50% *vs*. 30% of the dry matter weight) whereas males eat more blackberries (about 20% *vs.* 7% of the dry matter weight); in winter, females eat more bramble leaves (about 70% *vs.* 50% of the dry matter weight) whereas males eat more acorns (about 23% *vs.* 8% of the dry matter weight).

Clearly, the roe deer population of the Dourdan forest does exhibit inter-individual dietary differences between sexes and seasons, making this data set well-suited to determine whether the SSFA methodology can successfully identify intra-specific dietary variations from microwear structures.

## Results and Discussion

A first, overall two-way (sex and season) MANOVA points out a highly significant interaction between the two factors (Table 1 and 2). Thus, distinct one-way MANOVAs were run for each separate factor.

**Table 1:** Summary statistics (m mean and s.e.m. standard error of the mean) of molar microwear parameters for roe deer depending on the sexes and the seasons.

			Asfc		Smc		Hasfc			epLsar		Tfv		
		N	m	s.e.m.	m	s.e.m.	m	s.e.m.		m	s.e.m.	m		s.e.m.
Both sexes	all seasons	78	3.988	0.390	0.530	0.276	1.317	0.097		3.748	0.200	13932.8		719.7
females	winter	4	1.497	0.608	0.759	0.429	0.701	0.038		5.596	0.518	12390.9		4222.2
	spring	6	2.508	0.453	0.170	0.020	1.555	0.208		4.400	0.516	12703.7		2788.7
	summer	8	2.689	0.820	0.511	0.227	1.100	0.186		2.880	0.414	16671.6		2859.6
	autumn	11	3.930	0.835	2.133	1.947	1.828	0.496		3.745	0.474	14046.3		1510.3
males	winter	10	4.368	0.855	0.201	0.028	1.309	0.138		1.949	0.289	11233.2		1940.8
	spring	18	4.746	1.041	0.191	0.013	1.281	0.166		3.138	0.374	14557.7		1664.8
	summer	12	4.458	0.720	0.194	0.024	1.171	0.128		4.544	0.404	14580.1		2015.5
	autumn	9	4.740	1.872	0.220	0.031	1.274	0.346		5.425	0.620	13751.0		969.9

Asfc: complexity; Smc: scale of maximum complexity; Hasfc: heterogeneity of complexity; epLsar: anisotropy (multiplied by 10^3^); Tfv: total fill volume.

**Table 2:** Intra-population multivariate analyses of variances.

		Heteroscedastic variates	Lambda Wilk	*F*	*df*	*p*
Intra-population	Sex	Ø	0.888	1.658	5, 66	0.157
Intra-population	Season	Ø	0.808	0.977	15, 183	0.481
Intra-population	Sex*Season	Ø	0.533	3.120	15, 183	**<0.001**
Winter sample	males *vs.* females	Ø	0.233	5.259	5, 8	**0.019**
Spring sample	males *vs.* females	Ascf	0.672	1.751	5, 18	0.174
Summer sample	males *vs.* females	Smc	0.479	3.038	5, 14	**0.046**
Autumn sample	males *vs.* females	Ø	0.737	0.997	5, 14	0.454
Male sample	seasons *vs.* seasons	Ø	0.498	2.178	15, 114	**0.011**
Female sample	seasons *vs.* seasons	Smc	0.341	1.857	15, 58	**0.05**

Asfc: complexity; Smc: scale of maximum complexity.

### Sexual Differences Depending on Seasons

The one-way MANOVA highlighting sexual contrast shows significant differences between males and females in the winter and summer samples (Table 2). This is consistent with the ecological data summarized above. Indeed, Cransac et al [23] did not find any significant dietary differences between the sexes in spring and autumn but rather in winter and summer.

**Figure 2:**
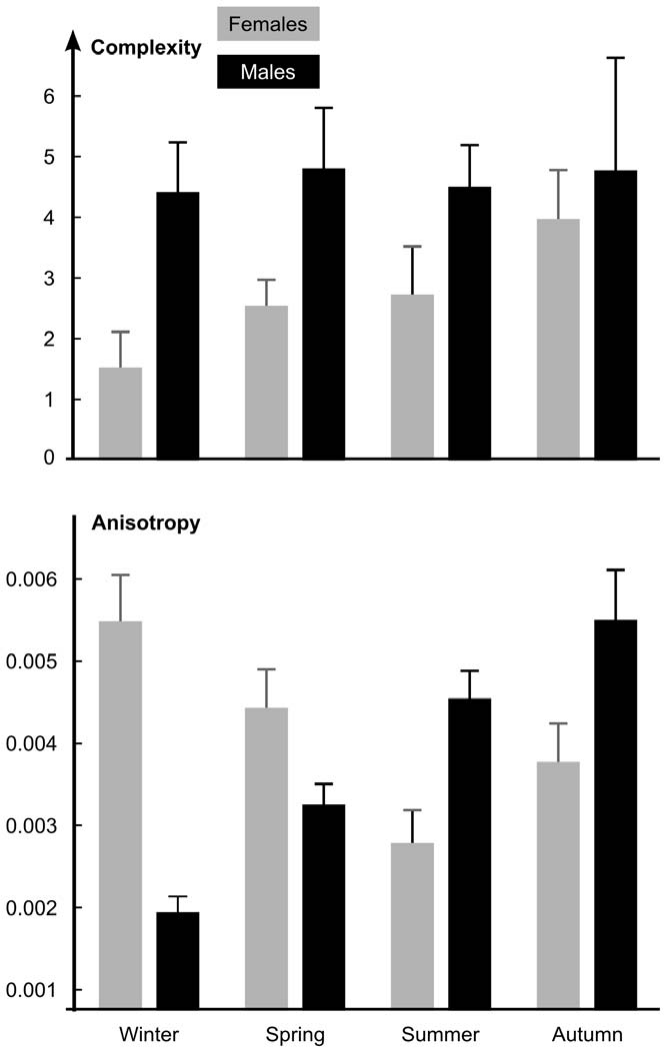
Molar microwear texture and intra-population variations in diet. The two variables, complexity and anisotropy (mean and standard error of the mean) show significant differences depending on season of death and sex. These results actually mirror the seasonal variations in leaf, fruit and seed availability and the feeding preference differences between males and females due to distinct energy requirements.

The different sets of one-way ANOVAs display significant differences in complexity (*Asfc*), anisotropy (*epLsar*), and heterogeneity (*Hasfc*) between males and females slaughtered in winter (Table 3). In fact, males shot in winter have higher complexity (*Asfc*) and heterogeneity (*Hasfc*) but lower anisotropy (*epLsar*) than females from the same season (Table 1, Table 2, Table 3, Table 4; Figure 2). Such textural differences between sexes might be linked to the higher intake of acorns by males during winter (23% *vs.*8% for females) and to the higher intake of bramble leaves (*Rubus sp*.) by females [23]. Furthermore, acorns are harder than foliage and consequently require greater pressure and more and longer chewing cycles during mastication.

**Table 3:** Intra-population univariate analyses of variances.

		Asfc		Smc		Hasfc		epLsar		Tfv	
	*df*	*F*	*p*	*F*	*p*	*F*	*p*	*F*	*p*	*F*	*p*
**males ** ***vs.*** ** females**											
winter sample	*1*	6.35	**0.027**	4.21	0.063	6.35	**0.027**	19.21	**<0.001**	0.07	0.790
summer sample	*1*	3.57	0.075	0.01	0.911	0.28	0.602	8.51	**0.009**	0.36	0.551
**seasons ** ***vs.*** ** seasons**											
male sample	*3*	0.34	0.795	0.35	0.789	0.41	0.748	10.61	**<0.001**	0.81	0.494
female sample	*3*	1.49	0.24	1.54	0.227	3.09	**0.045**	4.62	**0.010**	0.43	0.733

ANOVAs are conducted only if the MANOVAs results (Table 2) display overall significant differences among groups.

Asfc: complexity; Smc: scale of maximum complexity; Hasfc: heterogeneity of complexity; epLsar: anisotropy (multiplied by 10^3^); Tfv: total fill volume.

**Table 4:** Intra-population multicomparison tests.

Sex		male	male		male	male	female	female	female	female
	Season	winter		spring	summer	autumn	winter	spring	summer	autumn
male	winter									
male	spring	epLsar								
male	summer	**epLsar**		**epLsar**						
male	autumn	**epLsar**		**epLsar**	Ø					
female	winter	**Asfc Hasfc epLsar**								
female	spring			Ø			**Hasfc**			
female	summer				**epLsar**		**epLsar**	epLsar		
female	autumn					Ø	epLsar Hasfc	Ø	Ø	

These tests are conducted on variables that significantly vary among groups as shown in Tables 2 and 3. Bold characters mean that the significant differences are supported by both LSD (Least Significant Differences test of Fisher) and HSD (Honest Significant Differences test of Tukey) tests. Otherwise the normal letter is used for variables whose differences are only supported by the LSD test.

Asfc: complexity; Smc: scale of maximum complexity; Hasfc: heterogeneity of complexity; epLsar: anisotropy; Ø: no significant differences.

In summer, the only textural differences between sexes pertain to anisotropy *epLsar* (Table 1, Table 2, Table 3, Table 4). The males have higher values than the females. This might be linked to the higher intake of bramble leaves by females whereas males feed more on bramble fruits [23]. Besides the differences in their abundance, these dietary items (leaves and blackberries) have different physical properties and consequently do not impact the enamel surface in the same way. While the anisotropy *EpLsar* was seen as negatively correlated with the intake of acorns, the stronger pressure on blackberries by males compared to females has either no effect or an inverse effect. Compared to acorns, these multi-seeded fruits might not require substantial chewing cycles during mastication. In addition, it can also be hypothesized that, due to numerous hair-like thorns along the midrib on the underside of the leaf, bramble leaves need to be crushed before being swallowed. More chewing than expected would thus be needed for that plant when compared to other dicot foliages, leading to the unexpectedly low values observed for female anisotropy.

Provocatively, differences in microwear textures could also be linked to plausible variations in enamel hardness between males and females rather than simply to dietary differences. In the same way that osteoporosis weakens bone architecture, calcium deficiencies in dental tissue due to the metabolic investment in both pregnancy and lactation could weaken female tooth enamel. This is also hypothesized by a recent study [33] done on the hypselodont (ever-growing) incisors of twenty-four female rats divided into three equal groups: unmated, pregnant and post-lactating females. Even if the authors do not detect any significant variation in calcium and phosphate composition in the enamel tissue between the three groups of female rats, they mention (but without a corroborative test results) differences in scratch and crack densities on the enamel surface. Different arguments however dismiss this hypothesis. Contrary to bone, mature enamel is totally mineralized. Therefore, there is no chemical resorption and structural remodeling after maturation [34], [35]. Moreover, there is no obvious correlation between the number of pregnancies and intensive lactation on the one hand, and the physical state of the teeth on the other [36]. Finally, the hypothesis stating that enamel could be weakened as a consequence of physiological requirements appears unlikely and can be quite safely excluded. Therefore the differences in diet can be considered the most important factor impacting the molar microwear surface.

### Seasonal Differences Depending on Sex

Seasonal changes in diet are significant for both males and females. Acorns, for instance, are eaten more in autumn and winter and blackberries more in summer [23]. These seasonal differences are mirrored for both sexes through SSFA on molar microwear texture (Tables 2 and 3). Differences are limited to anisotropy (*epLsar*) for males and to anisotropy (*epLsar*) and heterogeneity (*Hasfc*) for females. There are significant seasonal differences for both sexes, with three exceptions: between autumn and summer for males, between autumn and summer for females and between autumn and spring for females (Tables 2 and 3; Figure 2). Males shot in spring have higher anisotropy (*epLsar*) than their winter relatives (Table 1, Table 2, Table 3, Table 4, Figure 2). Females shot in winter have significantly lower heterogeneity (*Hasfc*) than their spring and autumn relatives (Table 1, Table 2, Table 3, Table 4). Males shot in summer display higher anisotropy (*epLsar*) than their spring relatives whereas the value significantly decreases from spring to summer for females. As said above, there is no significant difference between summer and autumn for the samples of both sexes. However, a closer look reveals that the two main variables, complexity (*Asfc*) and anisotropy (*epLsar*), do increase from summer to autumn for both sexes (Table 1; Figure 2). The differences in microwear texture at the transition between autumn and winter are important. Males shot in winter have lower anisotropy (*epLsar*) than males slaughtered in autumn whereas the anisotropy of females shot in winter is higher than females shot in autumn. Besides this, the winter females also have lower heterogeneity (*Hasfc*) than the autumn females. However, the very small sample size for females shot in winter (*N* = 4) means we should be cautious about this result.

Given that microwear texture depends on the physical properties of each dietary item [4], differences were actually expected between seasonal samples (Table 1, Table 2, Table 3, Table 4). Nevertheless, according to the ecological data [23], lack of significant difference for males and females in microwear texture at the summer-autumn transition and the significant difference for males at the autumn-winter transition were both unexpected. In summer, roe deer browse on foliage and complete their diet with blackberries. These bramble fruits compose 20% of the male diet (expressed as the percentage of dry matter weight in the stomach contents) and about 7% of the female diet. In autumn, acorns compose the main source of food for both sexes, since they represent about 60% of the diet. Surprisingly, this strong dietary shift from summer to autumn is not significantly noted by the SSFA on molar microwear texture. The high amount of acorns in the diet of both males and females in autumn does not seem to affect their dental microwear texture when compared to the summer samples. Alternatively, acorns could impact the enamel surface in the same way as blackberries. However, while there is no evidence of a high amount of acorns in the male diet in autumn, their lower abundance in the male diet in winter is possibly detected by a lower anisotropy (*epLsar*).

Through experimental studies on captive primates with controlled foods, Teaford and Oyen [4] demonstrate that the switch between two significantly different microwear textures depends on the physical properties of the food. Hard items swiftly erase previous microwear scars in a few days, whereas the consumption of soft foods over a longer timespan is needed to reach the same result. Given the high taxonomical, anatomical, and mechanical disparity of the items composing the roe deer diet and their variations in abundance depending on sex and season, no clear evidence can be detected to explain these unexpected results, *i.e.* summer/autumn transition for both sexes and the autumn/winter transition for males.

### Individual-Scale Molar Microwear Texture and Stomach Contents Cross-Comparison

Cransac et al. [23] demonstrate that stomach contents analysis detects daily dietary variability between individuals (Table S1 and Table S2). Based on such stomach contents differences, one could expect that SSFA on molar microwear texture would detect inter-individual differences in daily diets.

The “two-block Partial Least-Squares” analysis (2b-PLS hereafter) of a data set of 58 individuals first allowed the “all-season & sex” question to be addressed: is there any “multivariate multiple linear covariation” between the five microwear texture variables on the one hand and the four main stomach contents variables on the other (*forbs*, *bushes/shrubs*, *bramble leaves*, and *acorns*)? Even if two synthetic axes are extracted from both sets of variables (blocks 1 and 2; Table 5) which together explain ∼92% of the identified covariance between the two blocks, the percentage of total possible squared covariance actually extracted is very small (0.89%). This indicates that these linear combinations of the original variables cannot be used in order to extrapolate the diet from microwear characteristics. Nevertheless, it is worth noting that the first two synthetic axes appear functionally meaningful. The first synthetic axis negatively associates anisotropy (*epLsar*) and surface complexity (*Asfc*) with *bramble leaf* consumption. The second synthetic axis negatively associates heterogeneity in surface complexity (*Hasfc*) with the relative abundance of acorns. A meaningful functional covariation structure between microwear and diet seems to emerge, but it is so weak that it has no apparent practical use in terms of food item prediction based on SSFA results.

**Table 5:** Numerical synthetic results of the two-block Partial Least-Squares analysis performed on the complete, “all-season & sex” data set (58 individuals).

			Axis 1	Axis 2	Axis 3	Axis 4
Eigenvalues			0.345	0.212	0.112	0.035
% of total covariance			67.0	25.2	7.1	0.7
PLS Loadings	Block 1	Asfc	0.551	−0.427	−0.319	−0.498
		Smc	0.005	0.002	0.445	0.379
		epLsar	0.700	−0.083	0.568	0.163
		Hasfc	−0.332	−0.900	0.110	0.209
		Tfv2	0.331	−0.016	−0.604	0.734
	Block 2	Forbs	0.068	−0.177	0.968	−0.162
		Bush shrubs	−0.358	0.317	−0.063	−0.876
		Bramble leaves	−0.801	0.355	0.195	0.441
		Oak acorns	0.476	0.861	0.142	0.107

Block 1: log-transformed dental microwear texture variables; Block 2: *clr*-transformed stomach contents variables. Percentage of total possible squared covariance: 0.89%.

In order to further explore the nature of the relationship between the two sets of variables, we performed one-way MANOVAs based on the coordinates of the 58 individuals in the first two synthetic planes returned by the overall 2b-PLS analysis (*i.e.*, axes 1 and 2 computed for the two blocks of variables; Table 5). A first MANOVA focused on differences between the sexes (Table 6), whereas a second one tested seasonal differences (Table 6). Results clearly indicate that roe deer males and females do not differ in their microwear texture *vs*. stomach contents relationship, but that significant differences do occur between seasons. Actually, a post hoc contrast analysis based on Hotelling's pairwise comparisons returned significant results for all but the [winter *vs*. spring] and [spring *vs*. summer] couples at the 95% confidence level (Table 6), indicating strong differences in food *vs.* microwear relationships between winter, summer and autumn, with spring intercalated between the winter and summer samples.

**Table 6:** Stomach contents/microwear texture individual-scale analysis.

**A**) Factor: Sex			
Wilks' Lambda =	0.906	Pillai Trace =	0.094
df1	4	df1	4
df2	53	df2	53
F	1.38	F	1.38
p(H_0_: no difference)	0.25	p(H_0_: no difference)	0.25
**B**) Factor: Season			
Wilks' Lambda =	0.456	Pillai Trace =	0.643
df1	12	df1	12
df2	135.2	df2	159
F	3.90	F	3.62
p(H_0_: no difference)	3.9×10^−5^	p(H_0_: no difference)	8.5×10^−5^
**C**) post hoc inter-season contrast analysis			
		*p*	*p*, Bonferroni corrected
Winter	Spring	0.15	0.90
Winter	Summer	1.6×10^−3^	9.7×10^−3^
Winter	Autumn	1.5×10^−3^	8.9×10^−3^
Spring	Summer	≥0.17	1
Spring	Autumn	1.8×10^−3^	0.011
Summer	Autumn	3×10^−4^	1.8×10^−3^

The one-way MANOVA results (A and B) are based on the coordinates of the analyzed individuals in the first two synthetic planes returned by the overall two-block Partial Least-Squares analysis (Table 4). Then, the post hoc inter-season contrast analysis is based on Hotelling's pairwise comparisons (C).

Seasons rather than sex are the primary source of variation in the food *vs.* microwear relationship. Hence the need to investigate the within-season variation. In other terms, is the very weak overall covariation between variables of microwear texture and stomach contents the only spurious first order consequence of distinct covariation structures between the two sets of variables from one season to another? The separate 2b-PLS analysis of each season returned higher total percentages of squared covariance (spring: 4.6%; summer: 5.1%; autumn: 5.8%; winter: 16.0%; detailed results not shown), but these values nevertheless remained quite low, indicating that even at the single-season level, no high correlations between the two sets of variables can be identified. This is probably due to high inter-individual variability in the microwear texture *vs*. stomach contents functional relationship. Finally, the lack of overall covariation between microwear texture and stomach contents variables emerges as the combined consequence of between-season differences and within-season inter-individual variability, ultimately precluding any simple and accurate prediction between the two sets of variables.

### Prospects and Limits

The results of the intra-population comparisons indicate significant effects of both seasonal and sexual factors on microwear texture. Indeed, the seasonal changes in diet and the sexual differences in energetic needs are mirrored through the scale-sensitive fractal analysis on molar microwear texture.

However, many points remain to be clarified. For instance, because bramble leaves are available throughout the year, they probably impact the enamel surface in different ways depending on the season. Indeed, we can presume that these leaves are tougher in winter since they are more impoverished in water and richer in cellulose than during spring and summer [27], [37], [38]. Also, the impact of fruits in the diet cannot be treated independently from that of seeds. Besides physical properties, the difference in seed size requires different mastication processes [37]. What is the impact of large *vs.*small seeds, of fresh *vs.* dry leaves? All these points may find answers through experimentation on captive specimens, such as first initiated by Teaford and Oyen [4].

The investigation of the relationship between stomach contents and microwear textures in roe deer could have provided results quite similar to those found by Teaford and Oyen [4]. In some ways it did, since the relationship between food microwear significantly varies between most seasons. This emphasizes the fact that, from one season to another, different types of food impact the enamel surface in distinct ways. However, the lack of a strong relationship between stomach contents and microwear texture could have been expected but needed to be tested. Indeed, stomach contents is composed of food items ingested over the last few hours whereas microwear textures record the physical properties of items consumed over a period of days, weeks or even, in some cases, months. In this context, experiments with captive animals and controlled feeding as proposed by Teaford and Oyen [4] remain the best alternative for understanding the genesis of dental microwear. However, such studies are unfortunately not expected to be of great help to paleontologists, since the differences in microwear texture between fossil individuals from a given species–provided they all derive from a single, ecologically homogeneous population–will remain difficult to interpret. This is because the season of death and the sex of the individual are unavailable in most of these fossil cases.

## Materials and Methods

### The Dourdan Forest and the Roe Deer Population

The Dourdan domain (48°19′N, 02°01′E, Ile de France, France) is a 900 ha forest of which 90% is deciduous, dominated oaks (*Quercus sessiliflora* and *Q. peduncalata*), and 10% is coniferous (*Pinus sylvestris*) [23]. The understory vegetation is mainly composed of brambles (*Rubus* sp.), hornbeam (*Carpinus betulus*), hawthorn (*Crataegus* sp.) and honeysuckle (*Lonicera peryclimenum*), while silver birch (*Betula verrucosa*), privet (*Ligustrum vulgare*), blackthorn (*Prunus spinosa*), ivy (*Hedera helix*), and holly (*Ilex aquifolium*) are minor species. Various forbs, mushrooms, mosses, and monocotyledons fill out the herbaceous vegetal formation [23].

From 1980 to 1990, the forested domain of Dourdan was devoted to the scientific study of roe deer in their environment, and therefore totally protected from any human activity, especially hunting. From 5 deer per 100 ha in 1980, their density increased to 25 individuals per 100 ha in 1988. The feeding, sexual and breeding behavior, as well as the social organization of this population were then studied [23], [39], [40], [41]. After this 10-year period, 120 individuals were slaughtered following strict procedures. All of them were shot within eight short periods from February 1989 to November 1990 (Table S1). The osteological collection is currently housed at the “Comportement et Ecologie de la Faune Sauvage” Research laboratory (INRA, Castanet-Tolosan, France). All stomach contents were frozen and analyzed for diet composition using a macroscopic examination of plant fragments [23]. The composition of the diet of each individual roe deer was expressed as the percentage of weight (dry matter) of each dietary item in a 100 g subsample of stomach contents.

### Material

First, among the original sample (*N* = 120), only individuals with active occlusal facets on the third permanent molar were selected. Therefore, 78 adult individuals were chosen for the intra-specific analyses (Table S1). From that, 20 roe deer were afterwards excluded for the individual-scale approach (Table S1) as their stomach did not contain enough material for a reliable estimation of the composition of their last meal.

### The Scale-Sensitive Fractal Analysis Protocol

Many protocols, from casting to quantification, are employed in the dental microwear analyses [19]. Here we consider the protocol of Scott et al. [22]. Data were collected on shearing facet 1 of the second upper and lower molars (Figure 1) [42]. A 100×140 µm area, represented by nearly 432,000 points, was scanned at the center of the dental facet using a Sensofar Plμ white-light scanning confocal microscope with a ×100 objective [21], [22]. These areas were then levelled using SolarMap Universal software, producing digital elevation models with a vertical sampling interval of 0.005 µm and a lateral sampling interval of 0.18 µm. Resulting data were analyzed with Toothfrax and SFrax software using a scale-sensitive fractal analysis (SSFA hereafter) (Surfract, http://www.surfract.com) [21], [22].

SSFA is based on the fractal geometry principle that a surface can look different when observed at different scales. Thus, a surface that appears smooth at coarser scales can be significantly rougher on finer scales. SSFA is applied to length profiles (length-scale analysis) and to three-dimensional surfaces (area-scale and volume-filling scale analyses). Five variables of microwear texture are used here to distinguish the different dental microwear texture: area-scale fractal complexity (*Asfc*), anisotropy (*epLsar*), scale of maximal complexity (*Smc*), textural fill volume (*Tfv*), and heterogeneity of complexity (*Hasfc*) [21], [22].

### Statistical Analyses

Two distinct sets of statistical tests are performed on molar microwear texture data in order to detect inter-individual variations.

Firstly, a two-way multiple analyses of variance (MANOVA) is conducted on the roe deer population with sex and season of death as factors [43], [44]. Because there is a significant interaction between these two factors (Table 2), each of them is independently treated through different sets of MANOVAs and ANOVAs (Tables 2 and 3). Then, post-hoc contrast analyses are performed combining the conservative Tukey's Honest Significant Difference test (HSD test hereafter) and the Fisher's Least Significant Difference multiple comparisons test (LSD test hereafter) (Table 4). Such a combination for pairwise comparisons counterbalances the effects of types I and II error rates [43], [44]. Because normality is not guaranteed, all variables were rank-transformed before running every sets of analyses [45]. For all these statistical tests, the null hypotheses stipulate that the samples have similar molar microwear textures. Levene tests of homogeneity of variances were computed prior to all MANOVAs and ANOVAs (detailed results not shown here). In all but three cases (*Ascf* for the inter-sex winter ANOVAs, and *Smc* for the inter-season female and inter-sex summer ANOVAs), analyses returned non significant results at the α = 0.05 significance level, suggesting overall inter-group homoscedasticity of the analyzed data (Table 2). Consequently, in all cases, the significant pairwise comparison results cannot in principle be interpreted as the spurious consequences of heteroscedastic data.

Second, we investigated the individual-scale relationships between the microwear texture variables and stomach contents data through two-block Partial Least-Squares analysis (2b-PLS) [46]. Departing from the raw data of stomach contents (Table S2), we first defined *D* = 5 food items (Table S1; *Forbs*, *Bushes/Shrubs*, *Bramble leaves*, *Acorns*, and *Others*, the latter representing <20% of the stomach contents in weight for 85% of the 58 analyzed individuals) in order to simultaneously optimize the signal/noise ratio and minimize the number of 0-values in the data set. A sixth food item, blackberries, was added in the analysis only for the summer sample, this item not being recorded for the three other seasons. This item is a fruit with small hard grains able to induce particular dental erosion. Then we applied the centered log-ratio transformation (*clr*) [47] to the compositional space made of the relative (*not* absolute) weights of the *D* food items. Use of this transformation eliminates computational shortcomings linked to proportions when using multivariate techniques such as 2b-PLS, due to the fact that a spurious correlation effect between variables is introduced by the unit-sum constraint when transforming absolute quantities into relative ones, making the covariance matrix of a compositional space singular [47], [48].

For each analyzed individual, the *clr*-transformation is the simple function:
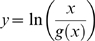
where
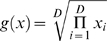
is the geometric mean to the compositional vector *x*. The use of the *clr*-transformation requires the previous replacement of 0-values–making *g(x)* = 0. Following Sandford et al. [49], each null value was replaced by a very small value (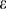) corresponding to 55% of the smallest possible relative abundance that can be obtained for a given individual, i.e.,
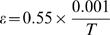
where *T* is the total weight of food items measured for the individual. Then, the *D xi*-values are proportionally readjusted to a unit sum, and the *clr*-transformation is finally performed.

Based on the five log-transformed microwear texture variables and *D*–1 *clr*-transformed stomach contents variables (all food items but the composite “*Others*” one, which does not convey a homogeneous signal in terms of dietary composition), we first performed a 2b-PLS analysis at the all-season level based on the forb, bush/shrub, bramble leaf and acorn food items, and then a separate analysis for each season. Two-block Partial Least-Squares is a “multivariate multiple covariation” procedure based on the singular value decomposition of the correlation matrix between two sets of original variables [46]. It can be viewed as a Principal Component Analysis, *but* with the objective of maximizing the covariation between two sets of variables treated symmetrically (here, microwear texture variables and relative abundance of stomach contents). Hence, the result of a 2b-PLS is two series (one for each set of variables) of new orthogonal axes (computed as the linear combination of the original variables and ranked in decreasing order of explained variance within each data set), defined so that the between-set covariance is maximized. Ultimately, the overall quality of the extracted covariance (*i.e.*, the adequacy of the least-squares approximation, equivalent to the usual Determination Coefficient in a classic bivariate linear correlation analysis) is calculated as the achieved percentage of total possible squared covariance between the two sets of synthetic axes.

Finally, we performed two one-way MANOVAs based on the coordinates of the analyzed individuals in the two synthetic spaces optimized for covariance. The goal here was to investigate the significance of the inter-individual sexual or seasonal differences, *taking into account* the identified structure of covariation between the two sets of original variables–which was not the case in the previous sets of analyses.

## Supporting Information

Table S1 Dental microwear texture parameters and stomach contents (weight of dry matter in g) in individuals investigated for individual scale analysis.
(0.15 MB DOC)

Table S2 Raw data from stomach contents expressed as weight (g) of dry matter.
(0.03 MB XLS)
