# Supplementary material for: Can Dental Microwear Textures Record Inter-Individual Dietary Variations?
Source: PLoS One. 2010 Mar 4;5(3):e9542. doi: 10.1371/journal.pone.0009542 (PMC2832010; doi:10.1371/journal.pone.0009542)
Supplement: Table S1 — Dental microwear texture parameters and stomach contents (weight of dry matter in g) in individuals investigated for individual scale analysis. (0.15 MB DOC) [file pone.0009542.s001.doc]

Table S1. Dental microwear texture parameters and stomach contents (weight of dry matter in g) in individuals investigated for individual scale analysis.

|  |  |  |  |  | dental microwear texture parameters | | | | | food categories used for the individual level analyses | | | | | | |
| --- | --- | --- | --- | --- | --- | --- | --- | --- | --- | --- | --- | --- | --- | --- | --- | --- |
| specimen | sex | season | d.o.d. | tooth | *Asfc* | *Smc* | *epLsar* | *Hascf* | *Tfv 2* | FI 1 | FI 2 | FI 3 | FI 4 | FI5 | others | Total |
| INRA 001 | f | wi | 01/02/89 | M2 | 0.837 | 2.015 | 0.00433 | 0.808 | 3203.5 | 0.018 | 0.051 | 0.269 | 0.000 |  | 0.000 | 0.338 |
| INRA 002 | f | wi | 02/02/89 | M2 | 1.223 | 0.268 | 0.00670 | 0.685 | 21919.5 | 0.003 | 0.149 | 0.855 | 0.000 |  | 0.005 | 1.012 |
| INRA 004 | m | wi | 03/02/89 | M2 | 1.896 | 0.150 | 0.00152 | 1.627 | 14027.5 | 0.007 | 0.165 | 2.005 | 0.000 |  | 0.001 | 2.178 |
| INRA 006 | f | wi | 07/02/89 | M2 | 0.643 | 0.600 | 0.00611 | 0.685 | 7823.3 | 0.000 | 0.140 | 0.611 | 0.000 |  | 0.002 | 0.753 |
| INRA 007 | m | wi | 08/02/89 | M2 | 1.991 | 0.417 | 0.00217 | 1.486 | 6458.4 | 0.009 | 0.057 | 0.189 | 0.000 |  | 0.013 | 0.268 |
| INRA 009 | m | wi | 09/02/89 | M2 | 2.691 | 0.151 | 0.00119 | 0.718 | 1461.4 | 0.000 | 0.093 | 1.622 | 0.000 |  | 0.002 | 1.717 |
| INRA 012 | m | wi | 13/02/89 | M2 | 2.011 | 0.153 | 0.00273 | 0.511 | 21169.4 | 0.126 | 0.438 | 0.547 | 0.000 |  | 0.155 | 1.266 |
| INRA 013 | m | wi | 13/02/89 | M2 | 4.862 | 0.151 | 0.00152 | 1.021 | 11053.5 | 0.000 | 0.019 | 0.753 | 0.000 |  | 0.000 | 0.772 |
| INRA 014 | f | wi | 13/02/89 | M2 | 3.285 | 0.152 | 0.00525 | 0.626 | 16617.2 | 0.015 | 0.098 | 0.468 | 0.000 |  | 0.000 | 0.581 |
| INRA 017 | f | sp | 05/04/89 | M2 | 0.750 | 0.152 | 0.00475 | 1.748 | 2933.7 |  |  |  |  |  |  |  |
| INRA 019 | m | sp | 10/04/89 | M2 | 1.985 | 0.150 | 0.00751 | 1.290 | 16180.4 | 0.077 | 0.231 | 0.880 | 0.000 |  | 0.028 | 1.216 |
| INRA 020 | m | sp | 11/04/89 | M2 | 9.766 | 0.150 | 0.00329 | 0.956 | 19699.5 | 0.022 | 0.282 | 0.241 | 0.000 |  | 0.032 | 0.577 |
| INRA 021 | m | sp | 12/04/89 | M2 | 1.047 | 0.152 | 0.00296 | 1.062 | 23792.3 |  |  |  |  |  |  |  |
| INRA 023 | m | sp | 17/04/89 | M2 | 6.708 | 0.160 | 0.00092 | 0.814 | 23046.2 |  |  |  |  |  |  |  |
| INRA 024 | m | sp | 18/04/89 | M2 | 5.730 | 0.267 | 0.00366 | 1.452 | 20067.9 |  |  |  |  |  |  |  |

INRA: Institut National de Recherche Agronomique; f: female; m: male; wi: winter; sp: spring; su: summer; au: autumn; d.o.d.: date of death; M2: second molars; *Asfc*: complexity; *Smc*: scale of maximum complexity; *epLsar*: anisotropy; *Hasfc*: Heterogeneity; *Tfv*: Texture fill volume. FI: Food item; FI 1: *Forbs* includes all herbaceaous dicot foliage, FI 2 *Bush/shrubs* includes all dicot foliage from shrubby, bushy and even arboreal formations, except *Brambles leaves* that compose their own category FI 3. Acorns FI 4 and Blackberries FI 5 compose their own categories. The difference in weight between the previous and the total weight of the stomach content is termed others.

Appendix 1 (continued)

|  |  |  |  |  | dental microwear texture parameters | | | | | food categories used for the individual level analyses | | | | | | |
| --- | --- | --- | --- | --- | --- | --- | --- | --- | --- | --- | --- | --- | --- | --- | --- | --- |
| specimen | sex | season | d.o.d. | tooth | *Asfc* | *Smc* | *epLsar* | *Hascf* | *Tfv 2* | FI 1 | FI 2 | FI 3 | FI 4 | FI5 | others | Total |
| INRA 025 | f | sp | 18/04/89 | M2 | 2.868 | 0.151 | 0.00532 | 1.556 | 14962.3 |  |  |  |  |  |  |  |
| INRA 026 | m | sp | 19/04/89 | M2 | 5.128 | 0.268 | 0.00096 | 2.065 | 9003.5 | 0.098 | 0.608 | 0.835 | 0.000 |  | 0.038 | 1.579 |
| INRA 029 | m | sp | 20/04/89 | M2 | 4.677 | 0.151 | 0.00212 | 1.626 | 17763.9 | 0.000 | 0.452 | 0.044 | 0.000 |  | 0.062 | 0.558 |
| INRA 031 | f | su | 08/08/89 | M2 | 1.213 | 0.150 | 0.00426 | 2.080 | 23065.3 | 0.203 | 0.153 | 0.182 | 0.000 | 0.000 | 0.001 | 0.539 |
| INRA 034 | m | su | 09/08/89 | M2 | 2.876 | 0.153 | 0.00494 | 1.144 | 3503.9 | 0.054 | 0.174 | 0.706 | 0.000 | 0.000 | 0.144 | 1.078 |
| INRA 035 | m | su | 10/08/89 | M2 | 4.241 | 0.151 | 0.00369 | 1.972 | 14846.9 |  |  |  |  |  |  |  |
| INRA 036 | m | su | 10/08/89 | M2 | 1.736 | 0.152 | 0.00555 | 0.636 | 27761.4 | 0.180 | 0.356 | 0.144 | 0 | 1.855 | 0.088 | 2.623 |
| INRA 037 | m | su | 11/08/89 | M2 | 6.138 | 0.153 | 0.00303 | 1.040 | 17782.8 | 0.513 | 0.058 | 0.811 | 0 | 0 | 0.008 | 1.39 |
| INRA 038 | f | su | 17/08/89 | M2 | 3.213 | 0.150 | 0.00121 | 1.486 | 27065.7 | 0.000 | 0.185 | 0.929 | 0.000 | 0.017 | 0.002 | 1.133 |
| INRA 039 | f | su | 18/08/89 | M2 | 0.880 | 0.267 | 0.00415 | 0.952 | 15089.7 |  |  |  |  |  |  |  |
| INRA 040 | m | su | 21/08/89 | M2 | 1.512 | 0.150 | 0.00436 | 1.208 | 15405.7 |  |  |  |  |  |  |  |
| INRA 041 | f | su | 22/08/89 | M2 | 5.605 | 0.151 | 0.00169 | 0.995 | 20251.1 | 0.108 | 0.334 | 0.747 | 0.000 | 0.359 | 0.019 | 1.567 |
| INRA 044 | m | su | 23/08/89 | M2 | 7.425 | 0.267 | 0.00286 | 2.031 | 17756.7 |  |  |  |  |  |  |  |
| INRA 045 | f | su | 23/08/89 | M2 | 1.075 | 0.600 | 0.00296 | 0.543 | 2010.8 |  |  |  |  |  |  |  |
| INRA 046 | f | au | 09/11/89 | M2 | 1.520 | 0.153 | 0.00505 | 1.146 | 13608.5 | 0.065 | 0.126 | 0.096 | 1.127 |  | 0.021 | 1.435 |
| INRA 048 | m | au | 15/11/89 | M2 | 5.041 | 0.267 | 0.00557 | 3.707 | 18171.1 | 0.087 | 0.188 | 0.529 | 0.000 |  | 3.640 | 4.444 |
| INRA 049 | m | au | 16/11/89 | M2 | 2.568 | 0.268 | 0.00358 | 1.135 | 12127.4 | 0.153 | 1.166 | 0.577 | 2.555 |  | 0.628 | 5.079 |
| INRA 051 | m | au | 17/11/89 | M2 | 2.207 | 0.153 | 0.00654 | 1.095 | 16513.8 | 0.211 | 1.114 | 0.014 | 0.000 |  | 3.019 | 4.358 |
| INRA 052 | m | au | 17/11/89 | M2 | 11.407 | 0.155 | 0.00273 | 2.028 | 10060.8 | 0.567 | 0.274 | 0.340 | 1.434 |  | 0.012 | 2.627 |
| INRA 055 | f | au | 21/11/89 | M2 | 3.093 | 0.152 | 0.00410 | 1.432 | 20299.2 | 0.000 | 0.078 | 0.485 | 0.004 |  | 0.016 | 0.583 |

Appendix 1 (continued)

|  |  |  |  |  | dental microwear texture parameters | | | | | food categories used for the individual level analyses | | | | | | |
| --- | --- | --- | --- | --- | --- | --- | --- | --- | --- | --- | --- | --- | --- | --- | --- | --- |
| specimen | sex | season | d.o.d. | tooth | *Asfc* | *Smc* | *epLsar* | *Hascf* | *Tfv 2* | FI 1 | FI 2 | FI 3 | FI 4 | FI5 | others | Total |
| INRA 057 | m | au | 22/11/89 | M2 | 1.421 | 0.151 | 0.00351 | 0.396 | 12077.2 | 0.302 | 0.920 | 0.664 | 0.114 |  | 0.030 | 2.030 |
| INRA 058 | m | au | 23/11/89 | M2 | 1.695 | 0.150 | 0.00815 | 0.488 | 10716.4 | 0.026 | 0.171 | 0.286 | 6.288 |  | 0.009 | 6.780 |
| INRA 059 | f | au | 23/11/89 | M2 | 7.885 | 0.267 | 0.00318 | 2.090 | 17135.6 | 0.021 | 0.748 | 0.609 | 0.000 |  | 4.389 | 5.767 |
| INRA 062 | m | wi | 06/02/90 | M2 | 1.853 | 0.150 | 0.00399 | 1.784 | 10222.9 |  |  |  |  |  |  |  |
| INRA 066 | m | wi | 09/02/90 | M2 | 7.697 | 0.155 | 0.00115 | 1.326 | 8558.8 | 0.351 | 0.427 | 0.017 | 0.625 |  | 0.112 | 1.532 |
| INRA 068 | m | wi | 13/02/90 | M2 | 9.252 | 0.267 | 0.00101 | 1.388 | 18340.0 | 0.002 | 0.245 | 0.053 | 1.560 |  | 0.018 | 1.878 |
| INRA 069 | m | wi | 14/02/90 | M2 | 5.195 | 0.150 | 0.00237 | 1.384 | 15738.5 | 0.027 | 0.210 | 0.151 | 0.588 |  | 0.554 | 1.530 |
| INRA 070 | m | wi | 21/02/90 | M2 | 6.233 | 0.268 | 0.00185 | 1.845 | 5301.8 | 0.037 | 0.159 | 0.787 | 0.218 |  | 0.072 | 1.273 |
| INRA 071 | m | sp | 02/04/90 | M2 | 0.986 | 0.266 | 0.00289 | 0.587 | 15691.4 | 0.090 | 0.502 | 1.047 | 1.724 |  | 0.204 | 3.567 |
| INRA 073 | m | sp | 03/04/90 | M2 | 2.501 | 0.150 | 0.00273 | 0.457 | 13377.0 | 0.452 | 0.585 | 0.021 | 0.635 |  | 0.063 | 1.756 |
| INRA 074 | f | sp | 03/04/90 | M2 | 2.479 | 0.268 | 0.00490 | 0.762 | 14859.3 | 0.000 | 0.047 | 0.075 | 0.000 |  | 0.000 | 0.122 |
| INRA 075 | m | sp | 03/04/90 | M2 | 1.426 | 0.267 | 0.00470 | 0.596 | 2766.8 | 0.009 | 0.955 | 0.198 | 0.000 |  | 0.107 | 1.269 |
| INRA 077 | m | sp | 05/04/90 | M2 | 18.625 | 0.161 | 0.00361 | 2.027 | 19479.7 | 0.602 | 0.423 | 0.108 | 0.000 |  | 0.066 | 1.199 |
| INRA 078 | f | sp | 05/04/90 | M2 | 1.800 | 0.151 | 0.00226 | 2.226 | 8334.6 |  |  |  |  |  |  |  |
| INRA 079 | m | sp | 09/04/90 | M2 | 1.586 | 0.150 | 0.00445 | 0.914 | 14049.5 |  |  |  |  |  |  |  |
| INRA 080 | f | sp | 09/04/90 | M2 | 3.838 | 0.150 | 0.00562 | 1.823 | 12010.2 |  |  |  |  |  |  |  |
| INRA 081 | m | sp | 09/04/90 | M2 | 2.941 | 0.152 | 0.00073 | 3.164 | 11465.2 | 0.260 | 0.348 | 0.187 | 0.000 |  | 0.118 | 0.913 |
| INRA 082 | f | sp | 10/04/90 | M2 | 3.314 | 0.151 | 0.00356 | 1.218 | 23122.1 | 0.027 | 0.135 | 0.336 | 0.000 |  | 0.075 | 0.573 |
| INRA 084 | m | sp | 10/04/90 | M2 | 0.603 | 0.267 | 0.00387 | 0.390 | 7926.1 | 0.147 | 0.533 | 0.252 | 0.000 |  | 0.011 | 0.943 |
| INRA 086 | m | sp | 13/04/90 | M2 | 8.002 | 0.150 | 0.00388 | 1.643 | 24566.9 | 0.027 | 0.537 | 0.081 | 0.000 |  | 0.014 | 0.659 |
| INRA 090 | m | sp | 18/04/90 | M2 | 1.428 | 0.154 | 0.00335 | 0.835 | 226.5 | 0.449 | 0.604 | 0.442 | 0.000 |  | 0.087 | 1.582 |

Appendix 1 (continued)

|  |  |  |  |  | dental microwear texture parameters | | | | | food categories used for the individual level analyses | | | | | | |
| --- | --- | --- | --- | --- | --- | --- | --- | --- | --- | --- | --- | --- | --- | --- | --- | --- |
| specimen | sex | season | d.o.d. | tooth | *Asfc* | *Smc* | *epLsar* | *Hascf* | *Tfv 2* | FI 1 | FI 2 | FI 3 | FI 4 | FI5 | others | Total |
| INRA 092 | m | su | 01/08/90 | M2 | 7.466 | 0.156 | 0.00593 | 1.248 | 11960.2 | 0.132 | 1.720 | 0.036 | 0.000 | 0.000 | 0.141 | 2.029 |
| INRA 093 | m | su | 02/08/90 | M2 | 7.664 | 0.154 | 0.00471 | 0.701 | 15226.6 |  |  |  |  |  |  |  |
| INRA 094 | f | su | 02/08/90 | M2 | 6.752 | 0.150 | 0.00224 | 1.400 | 21707.8 | 0.046 | 0.168 | 1.211 | 0.000 | 0.230 | 0.010 | 1.665 |
| INRA 095 | m | su | 02/08/90 | M2 | 1.382 | 0.418 | 0.00533 | 0.692 | 17716.3 |  |  |  |  |  |  |  |
| INRA 096 | f | su | 03/08/90 | M2 | 0.701 | 0.600 | 0.00250 | 0.793 | 9702.1 | 1.156 | 1.239 | 1.075 | 0.000 | 0.381 | 0.099 | 3.950 |
| INRA 101 | m | su | 08/08/90 | M2 | 4.752 | 0.150 | 0.00677 | 1.075 | 11676.4 | 0.013 | 0.343 | 0.275 | 0.235 | 0.000 | 0.064 | 0.930 |
| INRA 103 | m | su | 09/08/90 | M2 | 2.027 | 0.266 | 0.00202 | 1.255 | 1649.9 | 0.147 | 0.592 | 0.282 | 0.000 | 0.449 | 0.154 | 1.624 |
| INRA 104 | f | su | 10/08/90 | M2 | 2.076 | 2.022 | 0.00403 | 0.548 | 14480.0 | 0.205 | 0.507 | 1.752 | 0.000 | 0.000 | 0.161 | 2.625 |
| INRA 105 | m | su | 10/08/90 | M2 | 6.272 | 0.157 | 0.00535 | 1.050 | 19674.1 | 0.283 | 0.169 | 0.098 | 0.000 | 0.573 | 0.027 | 1.150 |
| INRA 106 | f | au | 08/11/90 | M2 | 6.718 | 0.151 | 0.00311 | 1.343 | 16367.1 | 0.120 | 0.949 | 0.375 | 3.555 |  | 0.431 | 5.430 |
| INRA 107 | f | au | 09/11/90 | M2 | 1.606 | 0.151 | 0.00376 | 0.741 | 11950.7 | 0.002 | 0.700 | 0.272 | 8.344 |  | 0.107 | 9.425 |
| INRA 108 | f | au | 09/11/90 | M2 | 5.641 | 0.151 | 0.00475 | 0.875 | 12481.7 | 0.024 | 0.467 | 0.133 | 7.114 |  | 0.031 | 7.769 |
| INRA 109 | f | au | 15/11/90 | M2 | 8.754 | 0.266 | 0.00408 | 5.580 | 14037.7 | 0.038 | 0.242 | 0.774 | 0.000 |  | 0.818 | 1.872 |
| INRA 112 | f | au | 22/11/90 | M2 | 2.016 | 0.150 | 0.00438 | 0.776 | 2575.9 | 0.172 | 0.534 | 0.311 | 6.143 |  | 0.080 | 7.240 |
| INRA 113 | m | au | 22/11/90 | M2 | 0.623 | 0.417 | 0.00716 | 0.714 | 13608.8 | 0.046 | 0.106 | 0.124 | 0.808 |  | 0.006 | 1.090 |
| INRA 114 | m | au | 23/11/90 | M2 | 0.924 | 0.150 | 0.00663 | 0.664 | 13262.1 | 0.307 | 0.578 | 0.400 | 2.893 |  | 0.219 | 4.397 |
| INRA 115 | f | au | 26/11/90 | M2 | 1.696 | 21.603 | 0.00643 | 0.889 | 15963.3 | 0.237 | 0.805 | 0.152 | 1.049 |  | 0.137 | 2.380 |
| INRA 117 | f | au | 28/11/90 | M2 | 2.530 | 0.267 | 0.00154 | 0.778 | 20173.0 |  |  |  |  |  |  |  |
| INRA 119 | m | au | 04/12/90 | M2 | 16.777 | 0.272 | 0.00496 | 1.240 | 17221.3 | 0.138 | 0.319 | 0.022 | 4.404 |  | 0.050 | 4.933 |
| INRA 120 | f | au | 06/12/90 | M2 | 1.774 | 0.150 | 0.00082 | 4.460 | 9916.1 |  |  |  |  |  |  |  |
